# Cost-effectiveness of a blended learning approach for maternal and child health professional development training in Ethiopia: a quasi-experimental economic evaluation

**DOI:** 10.1136/bmjph-2026-005316

**Published:** 2026-10-27

**Authors:** Gizachew Tadele Tiruneh, Robel Tezera, Bezawit Mesfin Hunegnaw, Ismael Ali, Biruk Bogale, Sarah Mekonnen, Nebreed Feseha, Tamar Chitashvili, Binyam Desta Fekadu, Wuleta Betemariam, Dessalew Emaway

**Affiliations:** 1 LEAP Center, University of Global Health Equity, Kigali, Rwanda; 2 JSI Research and Training Institute Inc, Addis Ababa, Ethiopia; 3 JSI Research and Training Institute Inc, Washington, District of Columbia, USA

**Keywords:** economics, Health Personnel, Public Health Practice

## Abstract

**Introduction:**

Ethiopia has integrated blended learning into the national continuous professional development system to address persistent gaps in in-service training access, cost and quality and strengthen primary healthcare providers’ capacity to deliver reproductive, maternal, newborn and child health (RMNCH) services. However, evidence on its effectiveness and cost-effectiveness remains limited, particularly regarding provider adherence, client experience and quality of care. Guided by implementation science principles, the Kirkpatrick framework and activity-based costing, we assessed the cost-effectiveness of blended learning for improving RMNCH service delivery.

**Methods:**

We conducted a quasi-experimental, post-test economic evaluation with a separate control group. Data were collected in October 2025 through facility audits, direct observations and exit interviews with providers trained through blended learning and untrained providers in control sites. The primary effectiveness outcomes were composite quality indices integrating adherence to evidence-based antenatal care (ANC) and family planning practices and client experience of care. Costs were estimated using activity-based costing from a health-system perspective. We assessed cost per trainee, incremental costs and savings and cost-effectiveness.

**Results:**

Blended learning improved ANC provision scores by 10.4 percentage points and family planning scores by 25.9 percentage points compared with controls (Cohen’s d=0.72 and 1.30 respectively; p<0.01). Client experience improved by 3.3 percentage points (Cohen’s d=0.36; p<0.01). Composite quality indices increased by 10.8 and 22.9 percentage points for ANC and family planning, respectively (Cohen’s d=0.64 and 1.35; p<0.01). Training cost US$262.81 per trainee with blended learning versus US$360.10 for face-to-face training, saving US$97.29 (27%). Blended learning was dominant for both ANC and family planning, combining lower costs with greater effectiveness.

**Conclusion:**

Blended learning improved RMNCH service quality while reducing training costs. Sustaining and scaling this approach will require investment in digital infrastructure, supportive implementation systems and health-worker capacity, alongside longer term monitoring to determine whether improvements in provider performance translate into population-level RMNCH outcomes.

**WHAT IS ALREADY KNOWN ON THIS TOPIC**

- Traditional face-to-face in-service training for reproductive, maternal, newborn and child health providers in low and middle-income countries is costly, difficult to scale and often disrupts service delivery, while evidence suggests that blended learning can achieve comparable learning outcomes. However, robust evidence on the cost-effectiveness of blended learning, particularly its impact on provider adherence to clinical standards, client experience of care and integrated quality of care, has remained limited, especially in sub-Saharan Africa.

**WHAT THIS STUDY ADDS**

- This study provides empirical evidence that a nationally implemented blended learning approach in Ethiopia improved the quality of antenatal care and family planning services, enhanced client experience of care and was cost-saving compared with traditional face-to-face training. Blended learning was dominant, delivering higher effectiveness at a 27% lower cost per trainee.

**HOW THIS STUDY MIGHT AFFECT RESEARCH, PRACTICE OR POLICY**

- These findings support institutionalising blended learning within national continuous professional development systems as a cost-effective and scalable strategy for strengthening primary healthcare. The study also demonstrates the value of linking economic evaluation with observed service quality and client experience, informing future research and policy decisions on digital health workforce investments in resource-constrained settings.

## Introduction

 Ensuring high-quality reproductive, maternal, newborn and child health (RMNCH) services remains a persistent challenge in many low and middle-income countries (LMICs), including Ethiopia. Despite significant gains over the past two decades, preventable maternal and newborn deaths remain unacceptably high, and quality of care has emerged as a major bottleneck to achieving further reductions.^1–3^ Evidence from multiple settings shows that poor provider competence, inconsistent adherence to clinical guidelines and suboptimal client experience contribute substantially to missed opportunities for lifesaving interventions.^4–6^ Strengthening the capacity of primary healthcare (PHC) providers is, therefore, central to improving service delivery, reducing preventable mortality and achieving universal health coverage.

Traditional face-to-face in-service training—though widely used—is often costly, difficult to scale and disruptive to service delivery, particularly in geographically dispersed or resource-constrained settings, because it requires extended provider absences, substantial logistical support and recurring expenses for travel, accommodation and venue rental.^7–10^ As a result, health systems in LMICs increasingly seek alternative training approaches that can expand access, reduce costs and maintain or improve learning outcomes. Digital innovations such as blended learning, which integrates digital self-learning with targeted in-person skills practice, has emerged as a promising modality for health workforce development globally.^11^ Studies from sub-Saharan Africa (SSA) and South Asia show that blended learning can achieve learning outcomes equivalent to or better than traditional face-to-face training, while offering greater flexibility, lower opportunity costs and improved scalability.^11–16^ Recent evidence from SSA further supports the potential of blended approaches for health worker training.^17,18^ In Ethiopia, a recent evaluation of a blended RMNCH refresher training for health extension workers reported a 39% reduction in recurring training costs compared with conventional training, with comparable knowledge outcomes and improved practical skills.^19^

Despite growing evidence, rigorous cost-effectiveness and cost-efficiency analyses of blended RMNCH training remain limited. Existing studies have focused mainly on training outcomes, feasibility, knowledge and skills, or direct training costs, with little evidence linking blended training to clinical service quality and client experience while assessing incremental cost-effectiveness.^18,19^ Evidence is also limited on how costs change as blended learning scales to larger trainee cohorts. This study addresses these gaps by evaluating a large-scale blended RMNCH training model in Ethiopia’s routine PHC system, linking training costs with observed ANC and family-planning quality and client experience and assessing cost-effectiveness and scalability relative to modelled face-to-face training.

The Ethiopian Ministry of Health (MOH) has prioritised digital learning through its national continuous professional development (CPD) platform to expand training access, standardise learning and sustain continuity.^20,21^ However, evidence on the costs and effectiveness of blended RMNCH training in routine health-system settings remains limited.

Since November 2023, the Gates Foundation–funded Strengthening Service Delivery (SSD) project, in partnership with the MOH and development partners, has implemented blended learning for priority RMNCH training. Guided by implementation science and the Kirkpatrick framework, this evaluation assessed the effectiveness and cost-efficiency of blended learning in improving provider competence and the quality of antenatal care (ANC) and family planning services. Using activity-based costing and a composite quality-of-care index, the study integrates measures of care provision, client experience and costs to generate evidence for national policy, scale-up decisions and broader efforts to strengthen the health workforce through digital and blended learning.

## Methods

### Context

The Ethiopian health system is restructured into three tiers: primary, secondary and tertiary care. The PHC tier is the foundation of the health system and typically comprises one primary hospital, 4–5 health centres and 20–25 health posts per woreda (ie, district), serving a population of approximately 100 000 people. PHC services encompass promotive, preventive, curative and rehabilitative care delivered through static facilities—including basic health posts, comprehensive health posts (upgraded health posts providing comprehensive RMNCH services, including delivery and outpatient services), health centres and primary hospitals—at home or in the community, and through outreach services.

Since November 2023, with support from the Gates Foundation, JSI has implemented the SSD project to improve PHC service quality and increase uptake of high-impact, integrated maternal and child health, family planning and immunisation services.^22^ The project focuses on three outcomes: strengthening provider competencies, optimising service delivery structures and processes, and enhancing last-mile delivery of essential supplies. Implementation follows a phased approach. The project initially rolled out in 36 woredas—28 agrarian and eight pastoral—across eight regions of Ethiopia (Afar, Amhara, Oromia, Sidama, Central, Somali, South and Southwest). By November 2025, an additional 14 agrarian woredas were enrolled, reaching the life-of-project target of 50 woredas, covering 1030 health posts, 257 health centres and 24 primary hospitals.

### Intervention description

To address gaps in provider competence, JSI, under the auspices of the MOH, designed a blended learning model that pairs self-paced eLearning modules with structured, competency-based face-to-face sessions. In January 2024, the project conducted a landscaping assessment to examine the national digital learning ecosystem, map RMNCH in-service training needs and review existing MOH eLearning and blended learning initiatives. The assessment identified several systemic challenges—fragmented learning management systems (LMS), duplication of courses, poor quality of eLearning courses, insufficient governance structures and weak coordination among implementing parties. At the same time, there was clear national demand for scalable blended learning solutions, expansion of CPD programmes and greater digital training opportunities for health workers. In response, the SSD project focused on strengthening national governance and coordination mechanisms, standardising blended learning design and development processes, consolidating LMS platforms, improving information technology infrastructure and building local capacity to support sustainable, MOH-led digital learning implementation.

Priority RMNCH thematic areas were identified based on national training needs and their potential to improve maternal, newborn and child health outcomes. These included family planning, ANC and preconception care (PCC), hypertensive disorders of pregnancy, postpartum haemorrhage management using E-MOTIVE, Integrated Management of Newborn and Childhood Illness, kangaroo mother care (KMC), community KMC, essential newborn care (ENC), immunisation in practice and the integrated pharmaceutical logistics system. The project supported the design, development, updating and revision of blended learning courses across these priority areas and produced a national blended learning design and development guide to standardise future implementation.

The development process followed a structured codesign model, involving content analysis, design matrix the creation, storyboard of development, interactive eLearning authoring using Articulate 360, multimedia content production and preparation of aligned face-to-face training materials. The blended curriculum consisted of competency-based modules compatible with MOH CPD requirements and designed for national scale-up through 15 public CPD centres. The family planning track consisted of two fully online modules—Basics in Family Planning and Short-Acting Methods (eight continuous educational units (CEUs)) and Client-Centered Counseling (8 CEUs)—as well as two hybrid modules that combined eLearning with hands-on clinical practice: Long-Acting Reversible Contraception (10 eLearning CEUs and 15 face-to-face CEUs) and Postpartum Family Planning (10 eLearning CEUs and 15 face-to-face CEUs). Maternal health modules, including ANC and PCC, were offered as balanced hybrid courses, each comprising 6 eLearning and six face-to-face CEUs. Between December 2024 and July 2025, the blended learning programme trained 2470 health workers—including nurses, midwives, health officers, general practitioners (GPs) and integrated emergency obstetric and surgical officers (IESOs)—in ANC/PCC, postpartum family planning (PPFP) and comprehensive family planning. Training was delivered via the MOH’s CPD eLearning platform and reinforced with competency-based face-to-face sessions at 14 public CPD centres.

### Study design

We conducted an economic evaluation using a quasi-experimental post-test design with a separate comparison group to assess the cost-effectiveness and cost-efficiency of a blended learning approach for priority RMNCH training in Ethiopia. Training effectiveness was measured by the application of acquired competencies in routine clinical practice and associated improvements in service quality, corresponding to the ‘Results’ level of Kirkpatrick’s evaluation framework.^23^ The analysis included both intervention facilities and comparison facilities that had not yet received the SSD intervention at the time of assessment but were scheduled to begin implementation in November 2025. Comparison woredas were selected based on similarity in facility characteristics and geographic context to the intervention woredas, and facilities were matched on key service delivery and readiness indicators.

### Study population and sampling

Using a stratified sampling approach, 95 facilities were assessed in October 2025, comprising 66 intervention and 29 comparison facilities. The sample included 14 comprehensive health posts and 11 primary hospitals, as well as 41 randomly selected health centres. Within each selected facility, data collection included observations of two providers, four paired client–provider consultations and exit interviews with ANC and family planning clients. Direct observations of client–provider interactions and exit interviews were conducted in both SSD intervention and comparison woredas. A total of 103 ANC and 116 family planning consultations were observed in intervention woredas, compared with 57 ANC and 57 family planning consultations in comparison woredas. Client care experiences were assessed through exit interviews with 240 ANC and family planning clients in intervention sites and 115 clients in comparison woredas during the postintervention period.

### Data collection and measurement

Data were collected in October 2025 through facility audits, direct observation of client consultations and exit interviews. Outcomes among providers trained through blended learning in intervention woredas were compared with non-trained providers in control areas that had not yet received blended training. The primary effectiveness outcome was a composite quality index capturing evidence-based ANC and family-planning practices and clients’ experience of care.

Facility readiness was assessed using structured audits covering general service readiness (basic amenities, equipment, infection prevention and diagnostic capacity) and service-specific ANC and family-planning readiness. Each domain was expressed as the percentage of tracer items meeting predefined criteria. Readiness measures were used to assess facility comparability and as potential confounders in multivariable analyses (online supplemental additional file 1).

Routine clinical encounters were observed using standardised checklists. ANC observations assessed history taking, examination, laboratory testing and counselling, while family-planning observations assessed clinical history, method counselling and side-effect discussion. Provision of care was measured using 31 ANC and 28 family-planning items. Exit interviews with the same clients assessed 13 experience-of-care items covering communication, dignity and respect and emotional support (online supplemental additional files 2–4). Internal consistency was acceptable (Cronbach’s α=0.78 for ANC, 0.71 for family planning and 0.70 for client experience).

Provision-of-care and experience-of-care scores were standardised to 0–100. A client-level composite quality index was calculated by multiplying the two scores, integrating technical quality and client-centredness. Scores were then aggregated to obtain mean quality indices for intervention and control sites.

### Economic evaluation

#### Costing approach

A health system perspective was adopted for the economic evaluation, and activity-based costing was used to capture all financial and opportunity costs associated with blended learning implementation. Financial and administrative records were reviewed to capture expenditures for a total of 2470 providers trained between March and July 2025 (eLearning plus face-to-face component for 1547): 69 on ANC and PCC, 697 on comprehensive family planning and 781 on PPFP. The costing framework included fixed costs, such as eLearning module design and LMS development, as well as recurring costs, including LMS maintenance, digital support, internet services, consumables and training materials. All LMS and programme design costs were amortised, and LMS costs were distributed across the 5500 trainees expected over the life of the project. Costs for face-to-face training delivery—facilitator fees, venue rental, travel, materials, equipment and practical session supplies—were also included.

Opportunity costs were calculated using national annual salary and benefit scales (Mid-level nurses, midwives and health officers: 114 000 ETB ≈ 850.7 US$; GP/IESO: 144 000 ETB≈1074.6 US$) and the time providers spent on eLearning and face-to-face sessions during working hours. Based on median completion patterns from LMS logs, approximately half of the eLearning time was estimated to occur during working hours.

Time requirements for blended learning were based on national training guidelines: ANC/PCC required 24 hours, comprehensive family planning 64 hours and PPFP 56 hours, with approximately half of eLearning time occurring during working hours. For comparison, a traditional face-to-face scenario was modelled using national curriculum durations: ANC/PCC 32 hours, comprehensive family planning 96 hours and PPFP 80 hours. Shared programme costs, such as supervision and logistics, were apportioned proportionally to training duration.

All 2024 costs were adjusted to 2025 Ethiopian Birr (ETB) using a consumer price index (CPI=1.0672). Given the 3-year project horizon and an expected 5500 trainees over the life of the project, costs occurring in subsequent project years were discounted at 3% to reflect the time value of money, consistent with commonly applied discounting conventions in health economic evaluations in LMICs. Costs were converted to US dollars at an exchange rate of 1 US$=134 ETB. The resulting cost per trainee for blended learning and traditional face-to-face models was compared with estimate total and per-provider cost savings. The comparative costing approach for the blended learning and face-to-face is presented in table 1.

**Table 1:** Comparative cost drivers and approach for blended learning and traditional face-to-face scenario

Cost domain	Blended learning	Traditional face-to-face scenario
Programme design and course development	Programme co-design, course revision, adaptation of eLearning and blended content	Programme codesign and course revision/adaptation workshops
LMS setup and infrastructure	One-time fixed costs (LMS, tablets, routers, printers, furniture); annualised over 3 years and distributed across 5500 life-of-project trainees	Not required (no LMS)
Supplies and equipment	Training materials, stationery, printing, medical supplies and consumables were amortised over 3 years	Scaled to training duration
Training delivery	Hybrid: eLearning hours included (6 hours. ANC/PCC, 26 hours. CFP, 10 hours. PPFP) + shorter face-to-face sessions (ANC/PCC 8 hours./1 day, CFP 48 hours./6 days, PPFP 40 hours./5 days)	Entirely face-to-face: longer duration (ANC/PCC 16 hours./2 days, comprehensive family planning 80 hours./10 days, PPFP 64 hours./8 days); higher facilitator, venue, travel and accommodation costs
Support and monitoring	TOT, eLearning trainee enrolment orientation and support posttraining follow-up	TOT and post-training follow-up only
Opportunity costs of provider time	Salaries valued for time away from routine services; time lost=24 hours. ANC/PCC, 64 hours. comprehensive family planning, 56 hours. PPFP; for eLearning, 50% of LMS ‘on-clock’ time included (3 hours. ANC/PCC, 13 hours. comprehensive family planning, 5 hours. PPFP)	Salaries valued for total face-to-face training time (32 hours. ANC/PCC, 96 hours. comprehensive family planning, 80 hours. PPFP); no eLearning component
Cost adjustment	2024 costs inflated to 2025 ETB (CPI=1.0672), discounted at 3%, converted at 1 US$=134 ETB	Same inflation, discounting and conversion applied

ANC, antenatal care; CFP, comprehensive family planning ; CPI, consumer price index; ETB, Ethiopian Birr; LMS, learning management systems; PCC, preconception care; PPFP, postpartum family planning; ToT, Training of Trainers.

Consistent with the activity-based costing approach, we organised all expenditures into specific cost domains, including initial fixed costs and recurring delivery and opportunity costs, to facilitate a head-to-head comparison, as detailed in table 1.

### Cost-efficiency and effectiveness analysis

Cost data were obtained from a separate, aggregate dataset capturing all financial and opportunity costs associated with both blended learning and traditional face-to-face training approaches. Blended learning costs were measured directly, while face-to-face training costs were estimated using blended learning and other programmatic data. The cost per trainee was calculated by dividing total expenditure by the number of trainees for each approach, with costs computed separately for each training modality and disaggregated by course type. Incremental cost savings and percentage reductions were then estimated to reflect system-level efficiency gains. A comparative cost-efficiency analysis was conducted to quantify the incremental savings of implementing blended learning for in-service ANC and family planning courses compared with traditional face-to-face training.

Cost-effectiveness was evaluated by linking the cost per trainee to the observed improvements in the composite quality index at the intervention and control sites. The incremental cost-effectiveness ratio (ICER) was calculated as the ratio of incremental cost to incremental effectiveness, comparing the blended learning intervention with control sites that had not received any blended training. This analysis combined the estimated incremental effect (blended learning outcome gain relative to control) with the difference in training costs between blended learning and no blended learning.

Furthermore, we applied a parity-effectiveness scenario, consistent with evidence showing that blended learning achieves comparable or superior outcomes to face-to-face training,^24,25^ since post-training outcomes for traditional face-to-face training were unavailable. Under this assumption, the ICER is determined solely by cost differences, allowing an assessment of whether blended learning is cost-saving relative to face-to-face training while delivering equivalent improvements in provider performance.

### Statistical analysis

Balancing tests using t-tests and χ² tests assessed comparability between intervention and comparison facilities. Facilities were broadly comparable in general readiness and other characteristics, although intervention facilities had higher ANC-specific and family planning-specific readiness scores (online supplemental additional file 5).

To address these imbalances, multivariable linear regression models were fitted for ANC and family-planning quality outcomes, adjusting for administrative region, provider professional category, number of technical staff and facility readiness characteristics, including amenities, equipment, infection prevention, diagnostic capacity and service-specific readiness. Adjusted mean differences with 95% CIs were estimated using Stata’s margins command.

Mean scores were compared using Student’s t-tests, with incremental differences representing the causal effect of the blended learning programme on provider competence and service quality. Cohen’s d was calculated to quantify the magnitude of differences between intervention and control groups, with values of approximately 0.2, 0.5 and 0.8 interpreted as small, moderate and large effect sizes, respectively.

### Sensitivity and scalability analysis

Scenario-based sensitivity analyses assessed the robustness of economic findings to key costing assumptions. The proportion of provider eLearning time occurring during working hours was varied from 30% to 70%, with 50% as the base case. Incremental costs and ICERs were recalculated for ANC and family planning under each scenario; blended learning was considered dominant when it was both less costly and more effective.

To assess economies of scale, unit costs were estimated under three trainee-volume scenarios: 2470 trainees (current implementation), 5500 (base case) and 10 000 (expanded scale).

## Results

### Trainee reaction, trainee knowledge improvement

Among the trainees who completed both pretraining and post-training knowledge assessments, mean knowledge scores significantly increased (p<0.01), indicating substantial learning gains across all ANC/PCC, comprehensive family planning and PPFP modules. Trainee feedback further demonstrated high acceptability of the blended learning approach, with 91% reporting satisfaction with the structure, content quality, and relevance of the courses. Participants also reported improved confidence and readiness to apply their new skills in routine service delivery.

Post-training surveys conducted with 2424 participants showed that the majority agreed or strongly agreed that the eLearning components were engaging, technically sound and well-integrated with face-to-face sessions. Behaviour assessments among trainees who completed ANC/PCC and family planning modules revealed high levels of self-reported application of learnt skills in clinical practice. Providers indicated that the blended learning approach strengthened their competency, enhanced adherence to evidence-based protocols and improved consistency in delivering high-quality care. Results from the face-to-face post-tests demonstrated significant improvements across all modules, with average scores increasing from below 70% at baseline to above 80% after face-to-face practice (figure 1).

**Figure 1:**
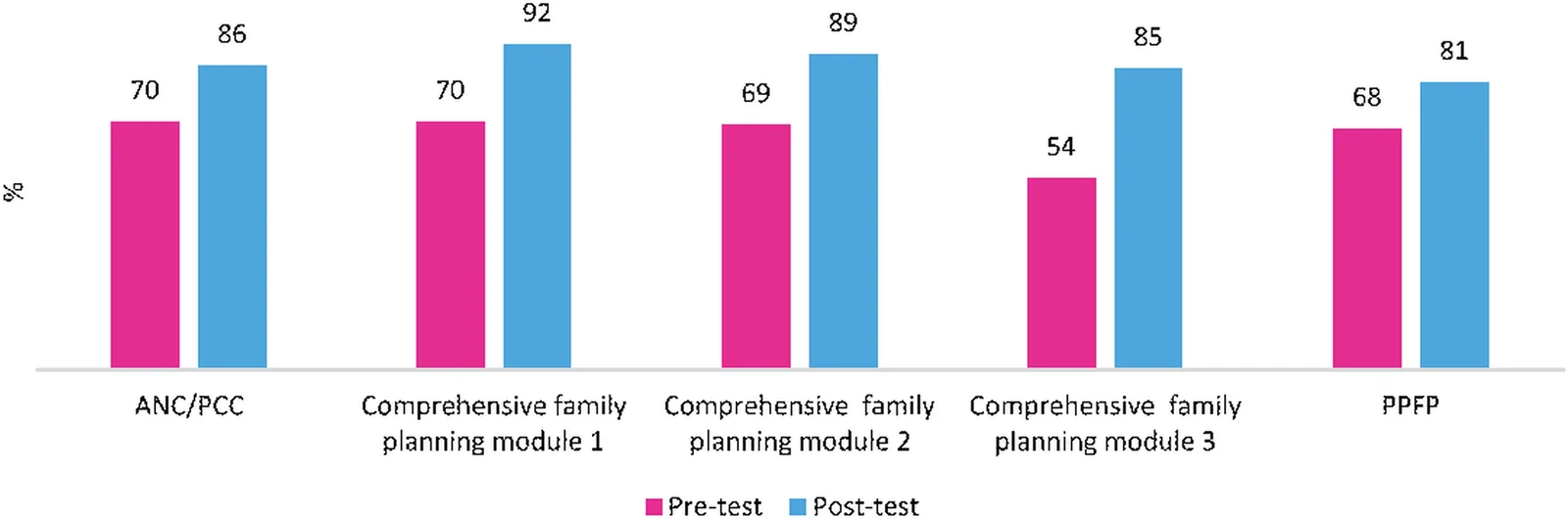
Pretest and post-test mean percentage score of blended learning participants, n=1700. ANC, antenatal care; PCC, preconception care.

### Clinical service quality and client experience quality of clinical service provision and client experiences of care

Table 2 presents quality-of-care provision scores for ANC and family planning services based on direct observations of client–provider interactions in intervention and control facilities. Providers who received blended learning training demonstrated significantly higher mean quality provision scores than those in control sites with no blended learning exposure. For ANC services, the mean quality provision index in intervention facilities was 77.1%, representing a 10.4–percentage-point improvement over control facilities (t=3.77; p<0.01; Cohen’s d=0.72, moderate effect). For family planning consultations, the improvement was substantially larger, with a 25.9–percentage-point difference between intervention (67.9%) and control facilities (42.0%) (t=8.19; p<0.01; Cohen’s d=1.30, large effect).

**Table 2:** Providers’ level of quality-of-care provision and client experiences of care in blended learning versus control sites, October 2025

Indicator	Service area	Study arm	Sample size	Mean percentage score	Incremental effect (%)	t value	P value	Cohen’s d
Quality service provision	ANC	Intervention	90	77.1	+10.4	3.767	<0.01	0.72
		Control	57	66.7
	Family planning	Intervention	111	67.9	+25.9	8.185	<0.01	1.30
		Control	55	42.0
Experiences of care	ANC/family planning	Intervention	201	83.0	+3.3	3.081	<0.01	0.36
		Control	112	79.7
Quality index	ANC	Intervention	90	64.0	+10.8	3.081	<0.01	0.64
		Control	57	53.2
	Family planning	Intervention	111	57.0	+22.9	7.895	<0.01	1.35
		Control	55	34.1

ANC, antenatal care.

Client-reported experience of care also improved significantly in blended learning intervention facilities, with a mean score of 83.0% compared with 79.7% in control facilities, corresponding to a 3.3–percentage-point increase (t=3.08; p<0.01; Cohen’s d=0.36, small to moderate effect).

### Overall quality index

Composite quality indices further demonstrated meaningful gains in intervention facilities, showing a 10.8–percentage-point improvement for ANC (t=3.08; p<0.01; Cohen’s d=0.64) and a 22.9–percentage-point improvement for family planning (t=7.90; p<0.01; Cohen’s d=1.35). Table 2 summarises these results.

After adjusting for potential confounders, the intervention was associated with significantly higher quality-of-care scores for both family planning and ANC. The adjusted mean quality score was 14.4 percentage points higher for family planning and 10.8 percentage points higher for ANC in the intervention group compared with the comparison group (table 3).

**Table 3:** Adjusted association between intervention status and quality of care outcomes

Outcome	Mean percentage score		Adjusted mean difference (95% CI)	P value
	Intervention	Comparison		
Family planning quality index	53.5	39.2	14.4 (7.7 to 21.0)	<0.001
ANC quality index	63.6	53.1	10.8 (3.6 to 17.4)	0.003

ANC, antenatal care.

### Training costs and cost savings

Cost analysis showed that implementation of the blended learning model was significantly less expensive than the traditional face-to-face model. The total cost per trainee under the blended learning model was US$262.81, compared with US$360.10 under the full face-to-face approach, yielding an absolute saving of US$97.29 per trainee, or a 27% reduction in total training expenditure.

Blended learning demonstrated substantial cost efficiencies across multiple cost drivers compared with traditional face-to-face training. It reduced face-to-face training delivery (40.6% savings), expenditure on supplies and equipment (40% savings) and provider salary opportunity costs (20.7% savings). However, programme design and course development incurred higher unit costs for blended learning due to initial development investments (US$5.87 vs 0.24), and LMS platform setup represented a unique fixed cost for the blended approach (US$18.71) (table 4 and online supplemental additional file 6).

**Table 4:** Cost per provider (US$) for blended learning and face-to-face, by cost driver

Cost drivers	Unit cost (US$)		Cost-saving (%)
	Blended learning	Traditional face-to-face	
Programme design and course development	5.87	0.24	−2345.83%
LMS platform setup	18.71	–	–
Supplies and equipment support	13.29	22.15	40.00%
Face-to-face training delivery	142.73	240.45	40.64%
Support and monitoring	15.55	13.23	−17.54%
Provider salary opportunity cost	66.66	84.03	20.67%
Total cost per trainee	262.81	360.10	27.02%

LMS, learning management systems.

Course-specific cost-saving estimates showed notable efficiencies for the comprehensive family planning and PPFP modules, with blended learning achieving savings of US$91–113 per trainee, representing 27%–29% cost reductions compared with traditional face-to-face delivery. For the ANC/PCC course, cost savings were more modest, at approximately US$5.04 per trainee (1.35%) (table 5).

**Table 5:** Cost savings for the blended learning and traditional face-to-face, by course

Course	Unit cost (US$)		Cost-saving (US$)	
	Blended learning	Face-to-face	Absolute saving	% point
ANC/PCC	368.85	373.88	5.04	1.35
Comprehensive family planning	279.51	392.58	113.06	28.80
PPFP	238.56	329.91	91.35	27.69
Family planning (comprehensive+PPFP)	246.38	343.45	97.07	28.26
Total (ANC/PCC+comprehensive family planning+PPFP)	262.81	360.10	97.29	27.02

ANC, antenatal care; PCC, preconception care; PPFP, postpartum family planning.

### Cost-effectiveness

The ICERs for ANC and family planning were negative, indicating that the blended learning approach is dominant, meaning it delivers equal or greater effectiveness at a lower cost.

For ANC, blended learning costs US$5.04 less per trainee face-to-face while achieving a 10.8 percentage point higher quality index. For family planning, blended learning costs US$97.07 less with a 22.9 percentage point higher quality index improvement. The resulting ICER values (table 6) indicated that blended learning provides higher quality at significantly lower cost. Under the parity scenario—where blended learning and face-to-face training are assumed to produce equivalent improvements in provider competence—the blended learning model was also found to be cost-saving and cost-effective.

**Table 6:** Cost-effectiveness analysis of blended learning vs traditional face-to-face

Course	Unit cost per trainee (US$)		Incremental cost (US$)	Intervention effect (%)		Incremental effectiveness (%)	ICER (US$ per % point)
	Blended learning	Face-to-face		Intervention	Control		
ANC	368.85	373.88	−5.04 (cost saving)	64.0	53.2	10.8	−0.47 (dominant)
Family planning	246.38	343.45	−97.07 (cost saving)	57.0	34.1	22.9	−4.24 (dominant)

ANC, antenatal care; ICER, incremental cost-effectiveness ratio.

Detailed supporting data for the cost-effectiveness analyses are provided in online supplemental additional file 7.

### Sensitivity of cost-effectiveness to key assumptions

Sensitivity analyses indicated that the cost-effectiveness findings were generally robust to variation in the assumed proportion of eLearning time occurring during working hours. For ANC, blended learning remained cost-saving and dominant at 30%, but became slightly more costly at 70%, with an incremental cost of US$5.85 per trainee and an ICER of US$0.54 per percentage-point improvement. For family planning, blended learning remained cost-saving and dominant across all scenarios (table 7).

**Table 7:** Sensitivity analysis: provider eLearning hours lost

Scenario	ANC		Family planning	
	Incremental cost (US$)	ICER	Incremental cost (US$)	ICER
Low (30%)	−15.92 (cost saving)	−1.47 (dominant)	−99.98 (cost saving)	−4.37 (dominant)
Base case (50%)	−5.04 (cost saving)	−0.47 (dominant)	−97.07 (cost saving)	−4.24 (dominant)
High (70%)	+5.85	+0.54	−94.16 (cost saving)	−4.11 (dominant)

ANC, antenatal care; ICER, incremental cost-effectiveness ratio.

### Scalability analysis: effect of trainee volume on unit costs

The scalability analysis demonstrated economies of scale, with overall unit costs declining from US$285.76 per trainee at 2470 trainees to US$262.81 at 5500 and US$254.15 at 10 000. Similar reductions occurred for ANC (US$391.92 to US$368.85 and US$359.73) and family planning (US$280.81 to US$246.38 and US$238.11), respectively.

## Discussion

Compared with the modelled conventional face-to-face training approach, the SSD-implemented blended RMNCH training strategy was estimated to be cost-saving while being associated with meaningful improvements in quality of care. The approach delivered high-quality care and improved client experience—particularly in adherence to ANC and family planning clinical standards. From a health system perspective, blended learning yielded a highly favourable ICER and reduced the cost per trainee by 27% relative to the traditional face-to-face model, while achieving substantial improvements in quality of care at minimal additional cost. These findings highlight the model’s strong potential for cost savings and position it as a scalable, sustainable training strategy for national expansion.

The blended learning was associated with substantial improvements in providers’ application of RMNCH competencies in routine clinical practice. This finding is consistent with evidence from other LMICs, showing that blended and eLearning approaches can achieve learning outcomes comparable to or better than conventional face-to-face training.^11,12,14–16,18,19^ By combining flexible, self-paced digital modules with structured hands-on clinical sessions and supportive learning systems, the approach strengthened providers’ ability to consistently apply evidence-based practices. These findings align with systematic reviews suggesting that blended learning can enhance provider competence through self-directed learning, repeated reinforcement, adaptable pacing and reduced cognitive overload,^11,12,24,25^ with potential implications for reducing preventable maternal and newborn morbidity and mortality.^26^

The blended learning model significantly improved women’s experiences of care, an increasingly recognised dimension of high-quality health systems.^3^ By integrating respectful maternity care, shared decision-making, privacy and empathic communication into the curriculum, the intervention strengthened client-centred care alongside clinical performance. These improvements contributed to gains in the composite quality index, which captures both technical and interpersonal dimensions of care, suggesting that blended learning translated improved provider competence into better client-perceived quality. This finding is consistent with evidence that digital and hybrid interventions incorporating respectful maternity care can improve provider attitudes, communication, and person-centred care behaviours.^27–30^

The intervention was cost-saving compared with the modelled traditional face-to-face training approach while achieving meaningful improvements in quality of care. The 27% overall cost reduction is broadly consistent with evidence from other LMICs, although savings vary by setting.^14,16,18,19^ Savings were primarily driven by shorter face-to-face training, lower travel and venue costs and reduced opportunity costs from minimising provider time away from service delivery. Similar studies show that blended and eLearning approaches can reduce training costs while maintaining or improving learning outcomes,^7,10,11^ with RMNCH programmes commonly reporting 20%–40% cost reductions^13,15^ and extended training programmes reporting savings of 65%–70%.^14,16^

Sensitivity analyses showed that the economic findings were generally robust but varied by course. For family planning, blended learning remained cost-saving and dominant across assumptions of 30%–70% of eLearning time occurring during working hours. For ANC/PCC, it remained cost-saving at 30% and 50% but became slightly more costly at 70%, indicating greater sensitivity to the opportunity cost of provider eLearning time. Scalability analysis demonstrated economies of scale as fixed LMS and curriculum-development costs were distributed across larger trainee cohorts, supporting the economic rationale for national scale-up.

Course-level differences in cost savings were largely driven by the balance between eLearning and face-to-face training. ANC/PCC generated modest savings because it included a greater proportion of in-person training, retaining costs for facilitators, venues, travel and other delivery requirements. In contrast, family-planning courses relied more heavily on self-paced eLearning, reducing resource-intensive face-to-face sessions and generating greater savings relative to conventional training. This is consistent with evidence from SSA showing that the economic advantage of blended learning is primarily driven by reductions in classroom time and associated travel, trainer and venue costs.^14,18^ Thus, the magnitude of savings depends substantially on the mix of online and in-person components.

Beyond direct training cost savings, the blended learning model also addressed persistent barriers to equitable access to in-service training and may generate additional health-system benefits by reducing prolonged provider absence from facilities. By allowing health workers in remote and underserved areas to complete the eLearning component asynchronously, the model may reduce the disruption to routine facility services and help maintain service continuity during training periods, potentially reducing patient waiting times and contributing to workforce retention in peripheral areas. The integration of blended learning within Ethiopia’s CPD framework further supports institutional sustainability and quality assurance, ensuring alignment with national health workforce strategies. In the Ethiopian context, where frequent staff turnover and geographic barriers limit access to traditional training, blended learning represents a practical, sustainable and scalable strategy.

This study has several strengths. It evaluated a blended RMNCH training model under routine health-system conditions and linked training costs with directly observed clinical service quality and client experience. The linked observation–exit interview design enabled assessment of both technical quality and client-centred care, while facility-level and provider-level characteristics were assessed for comparability and incorporated into multivariable analyses to reduce potential confounding. The economic evaluation additionally incorporated fixed and variable training costs, sensitivity analyses for key costing assumptions, and scalability scenarios to assess the implications of increasing trainee volumes.

Despite these strengths, several limitations should be considered when interpreting the findings. First, the post-test-only quasi-experimental design, absence of preintervention outcome measurements and unequal group sizes limit causal inference and do not exclude residual confounding from unmeasured baseline differences. Second, blended learning was implemented as part of the broader SSD package, including service integration and last-mile commodity strengthening; therefore, the independent contribution of blended learning cannot be fully separated from other SSD activities despite adjustment for measured facility-level and provider-level factors. Third, the effectiveness comparison was between intervention and not-yet-trained comparison facilities, whereas the economic analysis compared observed blended-learning costs with a modelled conventional face-to-face training scenario; these are, therefore, related but not identical comparators. Finally, the analysis focused on short-term provider performance and quality-of-care outcomes and did not estimate downstream maternal or neonatal health outcomes, broader societal costs or long-term benefits.

## Conclusion

In conclusion, the embedded blended learning model within the national CPD platform strengthened both the technical and experiential dimensions of RMNCH service quality while demonstrating strong cost-efficiency compared with conventional face-to-face training. By delivering quality outcomes comparable to traditional face-to-face training—but at substantially lower cost and with greater scalability—blended learning stands out as a viable and sustainable strategy for strengthening PHC in Ethiopia and other resource-limited settings.

Sustaining and scaling this approach nationally will require continued investment in digital infrastructure, supportive implementation systems and health-worker capacity, together with longer term monitoring to determine whether improvements in provider performance and service quality translate into meaningful population-level RMNCH outcomes.

## Supplementary material

10.1136/bmjph-2026-005316 online supplemental file 1

10.1136/bmjph-2026-005316 online supplemental file 2

10.1136/bmjph-2026-005316 online supplemental file 3

10.1136/bmjph-2026-005316 online supplemental file 4

10.1136/bmjph-2026-005316 online supplemental file 5

10.1136/bmjph-2026-005316 online supplemental file 6

10.1136/bmjph-2026-005316 online supplemental file 7

## Data Availability

All data relevant to the study are included in the article or uploaded as supplementary information.
