# Supplementary material for: Cost-effectiveness of a blended learning approach for maternal and child health professional development training in Ethiopia: a quasi-experimental economic evaluation
Source: BMJ Public Health. 2026 Oct 27;4(4):e005316. doi: 10.1136/bmjph-2026-005316 (PMC13723881; doi:10.1136/bmjph-2026-005316)
Supplement: Supplementary file 1 [file bmjph-4-4-s001.pdf]

Additional file 1: Facility readiness and quality index measures

Indicator	Definition and measurement
Family planning service readiness	**8 items:** injectables, oral contraceptive pills, male condoms, progestin-only pills, guidelines, job aids, blood pressure apparatus, and training on comprehensive family planning
ANC service readiness	**8 items:** blood pressure measuring machine, hemoglobin test, urine protein test, iron/folic acid tablets, injectable antibiotics, ANC guidelines, ANC job aid, and trained personnel on ANC
General service readiness	The general service readiness was measured using a set of tracer items audited within each facility to determine their presence on the day of the assessment.<br>1) **Basic amenities** are defined as reliable electricity for lighting and communication with no interruptions exceeding two hours per day over the past seven days, an improved water source, functional communication means, emergency ambulance transport availability, and internet connectivity.<br>2) **Basic equipment** refers to the presence of four essential items — an adult weighing scale, a child weighing scale, a blood pressure apparatus, and a thermometer — necessary for routine clinical assessment and basic patient care.<br>3) **Standard precautions** measures infection prevention capacity, including sterilization equipment, sharps disposal, and availability of infection prevention guide.<br>4) **Diagnostic capacity** is measured by access to basic lab tests: hemoglobin/hematocrit, blood group, urinalysis (protein and sugar), syphilis test, pregnancy test, malaria test (blood film/rapid), and HIV test for prevention of mother-to-child transmission.<br>5) **Essential medicines** audits the availability of a core list of primary care medications. The **general service readiness score** is the unweighted average of these five domain scores, providing a singular metric for overall facility preparedness.
Family planning service quality provision	Family planning service quality provision refers to the extent to which healthcare providers deliver client-centered, evidence-based, and comprehensive family planning services that ensure safe, informed, and effective contraceptive use. In this study, family planning service quality was assessed across three key domains:<br>1) Appropriate and pertinent history taking (7 items): collecting client information on age, last menstrual period, number of living children, last delivery date or age of youngest child, breastfeeding status, symptoms of sexually transmitted infections, and any chronic illnesses to guide safe and suitable method selection.<br>2) Appropriate and pertinent physical examination (6 items): measuring weight and blood pressure, auscultating the precordium, and inspecting/palpating the breasts to ensure the client is medically eligible for contraceptive methods.<br>3) Appropriate counseling and follow-up (15 items):<br>3.1. Method use counseling: providing instructions on method use, onset of effectiveness, duration, missed-dose guidance, discontinuation or

	removal options, method switching options, return-visit guidance, and use of visual counseling aids.<br>3.2. Side effects counseling: explaining expected bleeding changes, other common side effects, injection-related weight gain, IUCD-related cramps or acne, differentiating side effects from illness, emphasizing the temporary nature of side effects, managing irregular bleeding, and advising when to return if side effects occur.<br><br>Each item was scored based on whether it was appropriately addressed during the consultation. Domain-specific scores were calculated as the proportion of items completed, and an overall family planning service quality score was computed as the average across all 25 items, with higher scores reflecting higher adherence to quality family planning standards.
Quality ANC service provision	Quality ANC service provision refers to the extent to which healthcare providers deliver evidence-based, comprehensive, and standardized ANC services that address the physical, clinical, preventive, and counseling needs of pregnant women. In this study, quality ANC service provision was assessed using 31 items grouped into five domains:<br><br>1) Appropriate and pertinent history taken (8 items): Age of client, last menstrual period, gravidity/parity, gestational age, calculated, past obstetric history, present Pregnancy History, mental Health, and Medical and Surgical conditions<br>2) Physical examination (7 items): physical appearance, weight, blood pressure measured, height, conjunctiva for anemia, fundal height, fetal heartbeat after 20 weeks, presentation after 34 weeks, and to monitor maternal health status.<br>3) Diagnostic tests (7 items): urine testing, blood group, hemoglobin, HIV, VDRL, HBsAg, and ultrasound before 24 weeks completed to detect health conditions and guide clinical decisions.<br>4) Counseling (6 items): informing women about PMTCT, breastfeeding, family planning, birth preparedness, maternal nutrition, and harmful drugs to support informed decision-making and health-promoting behaviors.<br>5) Treatments and preventive care (3 items): provision of iron, deworming treatment, and tetanus toxoid vaccination to prevent maternal and neonatal complications.<br><br>Each item was assessed for completion during the ANC visit. Domain-specific scores were calculated as the proportion of recommended items completed, and an overall quality ANC service provision score was computed as the average across all 28 items, with higher scores indicating better adherence to ANC standards.
Experiences of care	Experiences of care refer to the ANC and family planning clients' perception of the interpersonal and supportive aspects of a healthcare encounter, reflecting how well the care experience meets the patient’s expectations for communication, respect, dignity, and emotional support. In this study, experiences of care were measured using 13 items grouped into three domains:<br><br>1) Effective communication (5 items): involvement in decisions about care, clarity of information and answers to questions, ability to ask questions, use of understandable language, and adequacy of consultation time.

	2) Respect and preservation of dignity (4 items): being addressed by name, being treated with respect, having opinions respected, and receiving adequate privacy during examinations.<br>3) Emotional support (4 items): provider responsiveness, comfort at the facility, and encouragement in decision-making.<br><br>Each item was scored on a standardized scale, and domain-specific scores were calculated as the average of items within each domain. A composite experiences-of-care score was computed by averaging all 12 items, providing a single summary measure of patient-reported experience during the healthcare encounter, with higher scores indicating better experiences of care.
Quality index	The quality index for ANC and family planning—encompassing both provision and experience of care—was calculated by multiplying provision-of-care scores with experience-of-care scores, derived from exit interviews and direct observation of consultations, and expressed as a percentage. Higher scores indicate greater adherence to quality standards and more favorable patient experiences.
