# Supplementary material for: Cost-effectiveness of a blended learning approach for maternal and child health professional development training in Ethiopia: a quasi-experimental economic evaluation
Source: BMJ Public Health. 2026 Oct 27;4(4):e005316. doi: 10.1136/bmjph-2026-005316 (PMC13723881; doi:10.1136/bmjph-2026-005316)
Supplement: Supplementary file 2 [file bmjph-4-4-s002.pdf]

Additional file 2: Observation (Client-provider Interaction) Tool

ID	Questions	Choices
region	Region	
woreda	Woreda	
hc	Health facility	
name	Name of data collector	
pname	Name of provider	
date	Date of interview	
q101	Provider category	1.Midwife 2. Nurse<br><br>3. Health officer
q102	Service observed	1. ANC<br>2. Family Planning
q103	Age of the mother	
q104	Number of prior deliveries client has had	
**general**	**Reception & Communication**	
q105	Greeted client politely and introduced self / role	1. Yes 0. No
q107	Asked client's reason for visit / chief complaint	1. Yes 0. No
q106	Explained purpose of consultation to client	1. Yes 0. No
q108	Allowed client to speak / asked open questions (listened without interruption)	1. Yes 0. No
**ipc**	**Infection Prevention**	
q109	Performed hand hygiene before physical exam / procedure (soap & water / sanitizer)	1. Yes 0. No
q110	Used gloves and aseptic technique when required (e.g., delivery, FP procedures, injections)	1. Yes 0. No
q111	Clean surface and safe waste disposal observed (sharps box, bio-waste)	1. Yes 0. No
anc_note	ANC consultation observation	
q112	**Appropriate and pertinent history taken**	
q112a	Age of client	1. Yes 0. No
q112b	Last menstrual period	1. Yes 0. No
q112c	Gravidity/parity	1. Yes 0. No
q112d	Gestational age is calculated	1. Yes 0. No
q112e	Past obstetric history	1. No 2. Yes 3. NA
q112f	Present Pregnancy History	1. No 2. Yes 3. NA
q112g	Mental Health	1. No 2. Yes 3. NA
q112h	Medical and Surgical	1. No 2. Yes 3. NA

q113	**Appropriate and pertinent physical examination**	
q113a	General appearance	1. Yes 0. No
q113b	Weight	1. Yes 0. No
q113c	Blood pressure	1. Yes 0. No
q113d	Conjunctiva is assessed for anemia	1. Yes 0. No
q113e	Fundal height (in wks.) is measured	1. Yes 0. No
q113f	Fetal heartbeat (after 20 weeks) is counted	1. No 2. Yes 3. NA
q113g	Presentation after 34 weeks is determined	1. No 2. Yes 3. NA
q114	**Appropriate investigation**	
q114a	Hemoglobin	1. No 2. Yes 3. NA
q114b	Blood group and RH	1. No 2. Yes 3. NA
q114c	VDRL	1. No 2. Yes 3. NA
q114d	HIV	1. No 2. Yes 3. NA
q114e	HBsAg	1. No 2. Yes 3. NA
q114f	Urine	1. No 2. Yes 3. NA
q114g	U/S before 24 weeks of gestation	1. No 2. Yes 3. NA 4. Referred
q115	**Appropriate treatment**	
q115a	Iron and folate	1. Yes 0. No
q115b	Td vaccine	1. No 2. Yes 3. NA
q115c	Mebendazole (Deworming medication)	1. No 2. Yes 3. NA
**q116**	**Appropriate counselling and follow up**	
q116a	Nutrition/healthy eating	1. Yes 0. No
q116b	PMTCT	1. Yes 0. No
q116c	Family planning	1. Yes 0. No
q116d	Breast feeding	1. Yes 0. No
q116e	Birth Preparedness and complication Readiness	1. Yes 0. No
q116f	Harmful drugs like alcohol, khat smoking, and other illicit drugs	1. Yes 0. No
q117	Gave clear follow-up instructions (next ANC date, where to go in emergency).	
**fp**	**Family Planning**	

cfp	Routine FP consultation	
q145	Assessed reproductive intention and method history (current use, reasons for switch).	1. Yes 0. No
q146	Screened for medical eligibility and contraindications (per WHO/ national checklist).	1. Yes 0. No
q147	**Appropriate and pertinent history taken**	
q147a	Age of client	1. Yes 0. No
q147b	Last menstrual period	1. Yes 0. No
q147c	Number of living children	1. Yes 0. No
q147d	Last delivery date or age of youngest child	1. Yes 0. No
q147e	Breastfeeding status	1. Yes 0. No
q147f	Symptoms of STIs (abnormal vaginal/urethral discharge)	1. Yes 0. No
q147g	Any chronic illnesses (heart disease, diabetes, hypertension, liver disease, or breast cancer)	1. Yes 0. No
q148	**Appropriate and pertinent physical examination**	
q148a	Weight	1. Yes 0. No
q148b	Blood pressure	1. Yes 0. No
q148c	Precordium auscultated	1. Yes 0. No
q148d	Breast is inspected and palpated	1. Yes 0. No
q148e	For IUCD insertion, bimanual palpation is done to determine size, position and signs of infection	1. Yes 0. No
q149	Provided balanced counseling on method options, benefits, and side effects.	1. Yes 0. No
q150	Modern methods of choice provided	1. Yes 0. No
q151	**Appropriate counselling and follow up: Method use**	
q151a	How to use the method or when to take the method	1. Yes 0. No
q151b	When the method becomes effective to prevent pregnancy	1. Yes 0. No
q151c	How long the method is effective to prevent pregnancy	1. Yes 0. No
q151d	What to do if forget to take the method on time for pills/ injectables	1. Yes 0. No
q151e	What to do if clients want to stop using or remove the method	1. Yes 0. No
q151f	Clients could switch to different methods if a selected method is not suitable after trying it	1. Yes 0. No
q151g	Return visit to resupply the method for pills, injectables, or condoms; to check the method for IUCD; to check pregnancy status for emergency contraceptives (EC)	1. Yes 0. No
q151h	Used any visual aids for health education or counselling	1. Yes 0. No
q152	**Appropriate counselling and follow up: Side Effects**	
q152a	Bleeding changes are common side effects (pills/injections/IUCD/implant)	1. Yes 0. No
q152b	Other side effects can occur such as headaches, dizziness, and breast tenderness(pills/IUCD/implant)	1. Yes 0. No
q152c	Weight gain(injection)	1. Yes 0. No
q152d	Abdominal cramps during monthly bleeding & acne (IUCD)	1. Yes 0. No
q152e	Side effects are not signs of illness	1. Yes 0. No

q152f	Most side effects usually become less or stop within the first few months	1. Yes 0. No
q152g	What to do to manage irregular bleeding such as taking pills regularly	1. Yes 0. No
q152h	The client can come back if side effects bother her or if she has other concerns	1. Yes 0. No
q153	Demonstrated method use where relevant (e.g., pill calendar, injectable schedule, condom demonstration).	1. Yes 0. No
q154	If a method provided: performed procedure safely (aseptic technique, correct administration).	1. Yes 0. No
q155	Gave clear instructions on follow-up and when to return/seek care.	1. No 2. Yes 3. NA
q156	Documented method and counseling in FP register/client card.	1. Yes 0. No
