# Supplementary material for: Cost-effectiveness of a blended learning approach for maternal and child health professional development training in Ethiopia: a quasi-experimental economic evaluation
Source: BMJ Public Health. 2026 Oct 27;4(4):e005316. doi: 10.1136/bmjph-2026-005316 (PMC13723881; doi:10.1136/bmjph-2026-005316)
Supplement: Supplementary file 3 [file bmjph-4-4-s003.pdf]

Additional file 3: Exit Interview (Experiences of Care) Tool

ID	Questions	Choices
region	Region name	
woreda	Woreda name	
hc	Health facility name	
name	Name of data collector	
date	Date of interview	
q101	q101. Client category	1.ANC 2. FP
q102	q1201a. How old were you at your last birthday?	
q103	q102. Have you ever attended school?	1. Yes 0. No
q104	q103. What is the highest level of school that you attended? (Probe: Did you attend primary, secondary, or higher?)	1.Primary<br>2.Secondary<br>3. Higher
q105	q104. What is the highest grade/ form/ year you completed at that level? Grade	
q106	q105. What is your current marital status?	1.Married<br>2. Single, never married<br>3. Divorced / separated / widow
q107	q107. What is your religion?	1. Orthodox 2. Protestant 3. Catholic 4.Other
q108	q108. How long would it take you to walk to this facility?	
q109	q109. How many times have you given birth so far?	
q110	q110. Is (was) the recent birth (or pregnancy if pregnant) wanted?	1. Yes 0. No
q111	q111. Estimated monthly income (in Birr)	
**exp**	**Overall experiences of care**	
q112	q112. Did the providers introduce themselves to you when they first came to see you?	1. Yes 0. No
q113	q113. Did the providers call you by your name?	1. Yes 0. No
q114	q114. How long did it take you from starting travel to health facility to reaching it (including time needed for acquiring transport)?	1.<15 minutes<br>2. 16-30 minutes<br>3. 31-45 minutes<br>4. 46-60 minutes<br>5. 60-90 minutes<br>6. 90-120 minutes<br>7. >120 minutes<br>99. Don’t know
q115	q115. About how long did you wait between the time you first arrived at this facility and the time you saw a staff for your consultation?	1. No waiting time<br>2. Reasonable/ short<br>3. Too long<br>88. Don't know
q116	q116. Do you feel that your waiting time was reasonable or too long?	1. Yes 0. No
q117	q117. Did the provider who attended you today use words that were easy to understand?	1. Yes 0. No
q118	q118. Do you think the time the provider spent in consultation with you was sufficient?	1. Yes 0. No
q119	q119. Did you feel the staff respected your opinions? Did the staff listen to your suggestions or opinions if you made them?	1. Yes 0. No
q120	q120. Did you have a pelvic exam during your visit today?	1. Yes 0. No

q121	q121. When you were being examined, did the provider explain to you what he/she was doing?	1. Yes 0. No 99. No response
q122	q122. Do you feel the information given to you during your visit today was too little, too much, or just about right?	1.Too little<br>2. Too much<br>3. About right<br>99. Don't know
q123	q123. Did you feel comfortable asking the provider questions?	1. Yes 0. No 99. No response
q124	q124. Did the provider clarify your concerns and answer your questions?	1. Yes 0. No 99. No response
q125	q125. Did you have enough privacy during your exam? (Probe: Clients or staff, other than those caring for you, could not see you.)	1. Yes 0. No 99. No response
q126	q126. When meeting with the provider during your visit, do you think other clients could hear what you said? (Note: This does not include the outside observer.)	1. Yes 0. No 99. No response
q127	q127. During your visit to the facility, how respectfully you treated by the provider? Would you say it is very respectful, respectful, disrespectful, or very disrespectful?	1.Very respectfully<br>2. Respectfully<br>3. Disrespectfully<br>4. Very disrespectfully<br>99. No response
q128	q128. During your visit to the facility, how respectfully were you treated by the other staff? Would you say it is very respectful, respectful, disrespectful, or very disrespectful?	1.Very respectfully<br>2. Respectfully<br>3. Disrespectfully<br>4. Very disrespectfully<br>5. There was no other staff<br>99. No response
q129	q129. In your opinion, how knowledgeable were the provider(s)? Would you say they are very knowledgeable, knowledgeable, not knowledgeable?	1.Very knowledgeable<br>2. Knowledgeable<br>3. Not knowledgeable<br>99. No response
q130	q130. Overall, how comfortable are you at the health center? Would you say you are very comfortable, comfortable, uncomfortable, or very uncomfortable?	1. Very comfortable<br>2. Comfortable<br>3. Uncomfortable<br>4. Very uncomfortable<br>99. No response
q131	q131. How responsive was the health center to your needs? Would you say it is very responsive, responsive, unresponsive, or very unresponsive?	1.Very responsive<br>2. Responsive<br>3. Unresponsive<br>4. Very unresponsive<br>99. No response
q132	q132. Did you feel like the provider at the facility involved you in decisions about your care?	0.No, never<br>1. Yes, all the time<br>2. Yes, most of the time<br>3. Yes, a few times<br>4. Don’t know/Can’t remember
q133	q133. Did you feel you understood the purpose of any tests you were asked to do? If yes, would you say this was all the time, most of the time, or only a few times?	1. Yes 2. No 3. NA
q134	q134. Did you feel you understood the purpose of any medicines you were given? If yes, would you say this was all the time, most of the time, or only a few times?	1. Yes 2. No 3. NA
q135	q135. Would you say you were treated poorly because of any personal attribute, like your age, marital status, language, number of children, your education, wealth, or	0.No<br>1. all the time<br>2. most of the time<br>3. a few times

	something like that? If YES, ASK: Would you say this was the case all the time, most of the time, or a few times?	
q135a	Did the doctors and nurses at the facility talk to you about how you were feeling?	1. Yes 0. No
q135b	When you needed help, did you feel the doctors, nurses or other staff at the facility paid attention?	1. Yes 0. No
q15c	Did you feel the doctors, nurses or other staff at the facility took the best care of you?	1. Yes 0. No
	**Does the provider use effective interpersonal communications?**	
q136	q136. Two-way communication	1. Yes 0. No
q137	q137. Use appropriate tone of voice	1. Yes 0. No
q138	q138. Listen attentively (eye contact, non-verbal cues, paraphrase/summarize patient concerns)	1. Yes 0. No
q139	q139. Uses appropriate visual aids or counseling tools	1. Yes 0. No
q140	q140. Encourage the client to talk (to ask questions or express feelings)	1. Yes 0. No
q141	q141. Does the provider encourage the client in making any decision or plan?	1. Yes 0. No
q142	q142. Does the provider ask about the presence of any general danger signs?	1. Yes 0. No
q143	q143. Does the provider inquire about the women to identify the woman's needs (woman's complains or any problem she might have)?	1. Yes 0. No
q144	q144. Did the provider seem caring and sympathetic to your needs or worries?	1. Yes 0. No
**q200**	**Experiences of care for FP clients**	
q201	q201. During your consultation today, did the provider: Ask about whether you would like to have a/another child?	1. Yes 0. No
q202	q202. During your consultation today, did the provider: Ask about when you would like to have a/another child?	1. Yes 0. No
q203	q203. During your consultation today, did the provider: Ask about your previous experience using FP?	1. Yes 0. No
q204	q204. During your consultation today, did the provider: Ask about your FP method preference?	1. Yes 0. No
q205	q205. During your consultation today, did the provider talk about possible side effects or problems with the method you selected?	1. Yes 0. No
q206	q206. During your consultation today, did the provider tell you what to do if you experience any side effects or problems with the method you selected?	1. Yes 0. No
q207	q207. During your consultation today, did the provider talk about warning signs associated with the method you selected?	1. Yes 0. No
q208	q208. During your consultation today, did the provider talk about the possibility of switching to another method if the method you selected was not suitable?	1. Yes 0. No
q209	q209. Did you feel that you received all of the information you wanted to know about your options for contraceptive methods?	1. Yes 0. No
q210	q210. During your consultation today, did the provider strongly recommend one method over others?	1. Yes 0. No
anc	ANC experiences of care	
q300x	ANC experiences of care	

q301	q301. Gestational age in weeks	
q302	q302. Which ANC visit (number) does the current visit represent?	
**q300**	**As part of your ANC during this ANC visit, were any of the following done:**	
q303	q303. Were you weighed?	1. Yes 0. No
q304	q304. Were you told the results after you were weighed?	1. Yes 0. No
q305	q305. What was your weight?	
q306	q306. Was your height measured?	1. Yes 0. No
q307	q307. Were you told the results of your height?	1. Yes 0. No
q308	q308. Was MUAC measured?	1. Yes 0. No
q309	q309. Record of MUAC in cm	
q310	q310. Was the provider give or prescribe food supplements such as Plumpy Nut or Super Cereal, if underweight	1. Yes 2. No 3. NA
q311	q311. Was your blood pressure taken?	1. Yes 0. No
q312	q312. Were you told the results after your blood pressure was taken?	1. Yes 0. No
q313	q313. Did you give a urine sample?	1. Yes 2. No 3. NA
q314	q314. Were you told the results of the urine test?	1. Yes 0. No
q315	q315. Did you give a blood sample?	1. Yes 2. No 3. NA
q316	q316. Were you told the results of the blood test?	1. Yes 0. No
q317	q317. If yes, please review the client's card and record the hemoglobin level or ask the mother if she remembers	
q318	q318. Was ultrasound taken?	1. Yes 2. No 3. NA
q319	q319. Was the provider listen to your baby's heartbeat?	1. Yes 0. No
q320	q320. Was the date of last menstrual period (LMP) recorded or asked?	1. Yes 0. No
q321	q321. Was the provider told you the expected date of delivery?	1. Yes 0. No
q322	q322. Are you screened for depression?	1. Yes 0. No
q323	q323. Do the provider give or prescribe antidepressants or referred to a mental health provider	1. Yes 0. No
q324	q324. Was the client given or prescribed calcium supplements?	1. Yes 2. No 3. NA
q325	q325. Was the client given a dose of TD vaccinations during any of the visits?	1. Yes 0. No
q326	q326. Was the client screened for danger signs?	1. Yes 0. No
q327	q327. Were you told about the signs of pregnancy complications?	1. Yes 0. No
q328	q328. Were you told where to go if you had any complications?	1. Yes 0. No
q329	q329. Did you experience any danger signs (such as vaginal bleeding, high blood pressure, convulsions, etc.) during the current pregnancy?	1. Yes 0. No
q330	q330. Did the provider manage or refer for specialized ANC at the hospital or with an obstetrician-gynecologist for the danger signs you experienced	1. Yes 0. No
q331	q331. Were you ever told how to monitor your baby's movements or to alert a health care provider if you felt fewer movements or no movement?	1. Yes 0. No
q332	q332. Were you ever told what to expect during your pregnancy and delivery?	1. Yes 0. No

q333	q333. Did a health provider ever talk to you about making prior arrangements for how to get to the health facility when you go into labor?	1. Yes 0. No
q334	q334. Did a health provider discuss things you should have in preparation for delivery? This may include planning in case of emergency, things you should bring to a facility or things you should prepare at home for this delivery.	1. Yes 0. No
q335	q335. Did a health provider ever talk to you about what to eat or how to eat well?	1. Yes 0. No
q336	q336. Did a health provider ever talk to you about iron with folic acid, how to take them, and any possible side effects?	1. Yes 0. No
q337	q337. Do providers provide or prescribe iron and folic acid ?	1. Yes 0. No
q338	q338. Do providers provide or prescribe any medicine for the treatment of intestinal worms (e.g., albendazole, mebendazole)?	1. Yes 0. No
q339	q339. Were you given any information or counseled about breastfeeding including early initiation within an hour and exclusive breast feeding till six months after delivery?	1. Yes 0. No
q340	q340. Were you given any information or counseled about exercise?	1. Yes 0. No
q341	q341. Did a health provider discuss any chronic illnesses such as diabetics, renal disease, cardiac disease, chronic hypertension, etc.?	1. Yes 0. No
q342	q342. Do you have any chronic illness? Or did the provider told you about the presence of any chronic illnesses?	1. Yes 2. No 3. NA
q343	q343. Did the provider give or prescribe medicine for the specific chronic illness or referred for specialized ANC at the hospital or with an obstetrician-gynecologist	1. Yes 0. No
q344	q344. Did a health provider discuss things you about prior obstetric complications such as previous surgery on reproductive tract, stillbirths, neonatal deaths, abortions, eclampsia, etc.	1. Yes 0. No
q345	q345. Did the provider manage or refer for specialized ANC at the hospital or with an obstetrician-gynecologist.	1. Yes 0. No
q346	q346. Has a provider advised you to use insecticide treated net (ITN)?	1. Yes 0. No
q347	q347. Has a provider given or prescribed you insecticide treated net (ITN) at any time during your visit?	1. Yes 0. No
q348	q348. During this or previous ANC visits for this pregnancy, did the provider talk to you about FP?	a. Yes, today<br>b. Yes, during the previous visits<br>c. No
q349	q349. Did the provider counsel you about options of FP methods?	1. Yes 0. No
q350	q350. Did the provider counsel you about PPFP methods?	1. Yes 0. No
q351	q351. During this or previous ANC visits for this pregnancy, did the provider talk to you about child vaccination?	1. Yes 0. No
q352	q352. Did the provider tell you when to return?	1. Yes 2. No 3. NA
q300x		
q601	q601. Were you told when to return for a follow-up visit?	1. Yes 0. No
q601a	q601a. Before you left, did the provider explain signs to watch for?	1. Yes 0. No
q602	q602. Were you satisfied with the advice or treatment that you received?	1.Very satisfied<br>2. More or less satisfied<br>3. Not satisfied

clean	Did you find this facility clean and welcoming?	
q603	q603. Clean	1. Yes 0. No
q604	q604. Welcoming	1. Yes 0. No
q604a	q604a. How clean were the toilets or latrines available to clients at the facility?	1. Yes 0. No
q605	q605. Are you planning to return here for next visit?	1. Yes 0. No
q606	q606. Would you recommend your neighbor to come to this facility?	1. Yes 0. No
q607	In general, which of the following statements best describes your opinion of the services you either received or were provided at this facility today?<br>READ ALL STATEMENTS, CIRCLE ONLY ONE	1. Yes 0. No
q608	q608. What did you like best about this facility?	a. Short waiting time to see provider<br>b. Short distance to facility<br>c. Facility is clean<br>d. Being treated with respect<br>e. Provider is competent/knowledgeable<br>f. Confidentiality/privacy<br>g. Good supply of medicines<br>h. Affordable cost of care<br>i. Access to ambulance<br>j. Being able to choose health care provider<br>k. Other<br>l. Don't know
q608_oth	Please specify other	
q609	q609. What suggestions do you have for improvement?	a. Shorten waiting time to see provider<br>b. Improve distance of health facility/open hospital around here<br>c. Clean the facility<br>d. Improve respect of providers toward patients<br>e. Improve skills of providers/assign doctors<br>f. Improve confidentiality/privacy<br>g. Improve supply of medicines<br>h. Reimburse costs incurred<br>i. Improve women's ability to choose a health care provider<br>j. Improve time required to get ambulance<br>k. Practice early referral<br>l. Avoid referral to hospital/treat all cases in this facility<br>m. Ambulance back home service<br>n. Other<br>o. Don't know
q609_oth	Please specify other	
**note4**	**Care seeking**	

q610	q610. In the last 12 months, how many times have you visited a health facility (including this one) for yourself for any reason?	
q611	q611. Did you see anyone for antenatal care for the previous pregnancy?	1. Yes 0. No
q612	q612. How many times did you receive antenatal care for the previous pregnancy?	
q613	q613. Where did you receive antenatal care for the previous pregnancy?	1. Yes 0. No
q614	q614. Where did you give birth in the most recent birth?	1. Yes 0. No
q615	q615. How much do you agree with the statement: I trusted that my care providers would keep me safe during this visit	1. Yes 0. No
q616	q616. Does the provider review suggested plan for follow-up care and adjusted plan to make it feasible in response to client's concerns?	1. Yes 0. No
q617	Now I would like you to think about your experiences with healthcare overall, not just for this visit	
q618	q618. Overall, how satisfied are you with the way the health care system works in your locality	0. I am not satisfied with the serviced I received<br>1. I am very satisfied with the services I received in the facility.<br>2. I am more or less satisfied with the services I received.
q619	q619. How confident are you that if you become very sick tomorrow, you would be able to receive effective treatment from the health system?	1. Yes 0. No
