# Supplementary material for: Cost-effectiveness of a blended learning approach for maternal and child health professional development training in Ethiopia: a quasi-experimental economic evaluation
Source: BMJ Public Health. 2026 Oct 27;4(4):e005316. doi: 10.1136/bmjph-2026-005316 (PMC13723881; doi:10.1136/bmjph-2026-005316)
Supplement: Supplementary file 4 [file bmjph-4-4-s005.pdf]

Additional file 5: General and service specific readiness

	Intervention	Comparison	X^2 or t (p-value)
Provider category			
Midwife	69.5	54.6	6.91 (0.032)
Nurse	22.3	33.6	
Health officer	8.1	11.8	
Administrative region			
Agrarian	100.0	70.2	41.36 (<0.01)
Pastoral	0.0	29.9	
Facility type			
Comprehensive health post	14.4	0.0	41.25 (<0.01)
Health center	64.2	96.4	
Primary hospital	21.4	3.6	
Technical staff density (per 10,000 population)	9.7	9.8	0.04 (0.965)
General service readiness			
Basic amenities	61.3	59.3	0.82 (0.408)
Basic equipment	90.8	87.0	1.91 (0.057)
Standard precautions	72.7	83.3	-2.10 (0.037)
Diagnostic capacity	88.1	92.6	-2.22 (0.028)
Overall general readiness	82.5	81.4	0.566 (0.576)
Service specific readiness			
Family planning	89.3	80.2	4.81 (<0.01)
ANC	80.1	70.0	4.99 (<0.01)
