# Supplementary material for: Cost-effectiveness of a blended learning approach for maternal and child health professional development training in Ethiopia: a quasi-experimental economic evaluation
Source: BMJ Public Health. 2026 Oct 27;4(4):e005316. doi: 10.1136/bmjph-2026-005316 (PMC13723881; doi:10.1136/bmjph-2026-005316)
Supplement: Supplementary file 5 [file bmjph-4-4-s006.pdf]

Additional file 6a: Cost per provider (USD) for blended learning, by courses and cost driver

	ANC (n=245)	CFP (n=2,478)	PPFP (n=2,777)	Total cost (USD) (n=5,500)	Cost per provider (USD)	
**Program design and course development**	1,440.12	14,547.31	16,300.50	32,287.93	5.87	2.2%
**LMS platform setup**	4,588.99	46,355.48	51,942.08	102,886.55	18.71	7.1%
**Supplies and equipment support**	-	34,499.27	38,592.41	73,091.68	13.29	5.1%
**F2F training delivery**	20,527.65	387,594.12	376,911.50	785,033.26	142.73	54.3%
**Support and monitoring**	3,815.28	38,539.81	43,184.49	85,539.58	15.55	5.9%
**Provider salary opportunity cost**	59,995.03	171,096.93	135,544.32	366,636.27	66.66	25.4%
**Total Cost**	90,367.06	692,632.92	662,475.30	1,445,475.27	262.81	100%
**Unit cost**	**368.85**	**279.51**	**238.56**	**262.81**		

Additional file 6b: Cost per provider (USD) for face-to-face, by courses and cost driver

	ANC (n=245)	CFP (n=2,478)	PPFP (n=2,777)	Total cost (USD) (n=5,500)	Cost per provider (USD)	
**Program design and course development**	59.60	602.78	675.51	1,337.89	0.24	0.1%
**Supplies and equipment support**	-	57,444.08	64,375.38	121,819.46	22.15	6.2%
**F2F training delivery**	17,118.00	667,811.75	637,518.38	1,322,448.13	240.45	66.8%
**Support and monitoring**	3,318.56	33,633.02	35,833.86	72,785.45	13.23	3.7%
**Provider salary opportunity cost**	71,105.22	213,315.65	177,763.04	462,183.90	84.03	23.3%
**Total Cost**	91,601.38	972,807.28	916,166.17	1,980,574.83	360.10	100%
**Unit cost**	**373.88**	**392.58**	**329.91**	**360.10**		
